# Perinatal Outcomes of Intrauterine Interventions for Fetal Sacrococcygeal Teratoma Based on Different Surgical Techniques—A Systematic Review

**DOI:** 10.3390/jcm13092649

**Published:** 2024-04-30

**Authors:** Hiroko Konno, Oluwateniayo O. Okpaise, Lourenço Sbragia, Gabriele Tonni, Rodrigo Ruano

**Affiliations:** 1 Division of Perinatology, Fetal Diagnosis and Therapy, Maternal and Perinatal Care Center, Seirei Hamamatsu General Hospital, Hamamatsu 430-0906, Japan; hxk565@miami.edu; 2 Medway Maritime Hospital, Gillingham ME7 5NY, UK; teniayookpaise@gmail.com; 3 Division of Pediatric Surgery, Department of Surgery and Anatomy, Ribeirao Preto Medical School, University of Sao Paulo, Ribeirão Preto 14049-900, SP, Brazil; sbragia@fmrp.usp.br; 4 Prenatal Diagnostic Centre, Department of Obstetrics and Neonatology, Istituto di Ricovero e Cura a Carattere Scientifico (IRCCS), AUSL Reggio Emilia, 42122 Reggio Emilia, Italy; gabriele.tonni@ausl.re.it; 5 Division of Maternal-Fetal Medicine, Department of Obstetrics, Gynecology & Reproductive Sciences, University of Miami, Miami, FL 33136, USA

**Keywords:** sacrococcygeal teratoma, fetal tumors, prenatal diagnosis, ultrasound, fetal surgery, fetal intervention

## Abstract

**Background:** This study aims to evaluate the outcomes of fetal sacrococcygeal teratoma (SCT) submitted to prenatal interventions. **Methods:** We performed a systematic literature review of fetal SCT patients and compared the outcomes between open fetal surgery and percutaneous intervention. In addition, we also compared the results of SCT fetuses who did not undergo any surgical intervention (NI). **Results:** We identified 16 cases of open fetal surgery (OS), 48 cases of percutaneous fetal intervention (PI), and 93 NI patients. The survival rate was 56.2% in OS, 45.8% in PI (*p* = 0.568), and 71.0% in NI patients. The gestational age at delivery was earlier in cases where there was no survival compared to cases where the fetuses did survive across all evaluated cohorts (OS: *p* = 0.033, PI: *p* < 0.001, NI: *p* < 0.001). The gestational weeks at delivery in OS and PI fetuses were more similar; however, OS tended to be performed later on in pregnancy, and the affected fetuses had more severe presented findings. In our evaluation, we determined that the presence of fetal hydrops and cardiac failure had no significant impact on survival in SCT cases. In NI patients, polyhydramnios was much higher in fetuses who did not survive compared to their surviving cohorts (*p* < 0.001). **Conclusions:** In conclusion, gestational age at delivery can affect the short-term prognosis of fetuses affected with sacrococcygeal teratomas. Regardless of the mode of delivery or the necessity for intervention during the fetal period, monitoring for complications, including polyhydramnios, can prevent premature delivery.

## 1. Introduction

Teratomas are neoplasms derived from the totipotent somatic stem cells in all the fetal germ cell layers, potentially allowing for a myriad of soft-tissue structures to form [1]. While teratomas typically develop in the gonads, they can develop at any level of the midline from the pineal gland to the coccyx; sacrococcygeal teratomas (SCTs) are the most common extragonadal location, particularly in fetuses and neonates [2].

SCTs affect approximately 1 in 20,000–40,000 live births, with a solid female predominance noted [3,4,5]. Sacrococcygeal teratomas can be divided into three histological categories: immature, mature, and malignant. Mature and immature SCTs are considered to be benign tumors and make up the majority, roughly 60%, of sacrococcygeal teratomas; however, fetal intervention should be considered [6].

Close monitoring of affected fetuses should be considered, as life-threatening complications can be associated with any SCTs as they are rapidly growing and highly vascular; these include fetal hydrops, cardiac failure, and polyhydramnios. This monitoring is achieved with serial ultrasound scans to determine the tumor volume-to-fetal weight ratio (TFR), a predictive tool for fetal outcomes [1,7]. As such, different interventions have been considered for SCT management, ranging from open surgery to minimally invasive procedures.

In fetuses with a gestational age greater than 28 weeks, elective delivery can be offered. In contrast, in fetuses of younger age who are at high risk of intrauterine hemorrhage or vascular steal, surgical procedures are necessary for fetal survival [8].

The use of open fetal surgery for SCT extraction was described over 30 years ago; it involves the use of anesthesia on both mother and fetus. Maternal laparotomy and uterine incision are performed, allowing for the visualization of the teratoma; the lower extremities are then delivered from the uterus, allowing the surgical team to have ample room to debulk and resect the SCT [8,9]. Following removal, Lactated Ringer’s solution is used to replace the amniotic fluid volume, the uterine wall and maternal abdomen is sutured, and tocolytics is administrated to prevent premature uterine contractions [9]. Minimally invasive procedures, such as fetoscopic laser ablation, can be preferred over open surgery as they cause considerably fewer impediments, making this a viable option in patients for whom open surgery is contraindicated or not a desired option for the mother [9]. Percutaneous radiofrequency ablation (RFA) is performed under ultrasonographic guidance and involves placing an 18-gauge needle containing insulated wiring to occlude feeding vessels, helping to reduce the tumor load and prolong the formation of cardiac failure [9,10]. However, fetal trauma can occur secondary to the thermal energy used to coagulate the SCT vessels; electrolyte abnormalities, including hyperkalemia, can occur as the tumor metabolites enter the fetal circulation [11]. Hence, there are no preferred therapeutic options, as advantages and disadvantages exist with both.

This study aims to systematically review different fetal interventions for sacrococcygeal teratomas. We will specifically focus on the indications for intervention, surgical technical aspects, and overall fetal outcomes.

## 2. Materials and Methods

This review was conducted according to the preferred reporting items for systematic reviews and meta-analyses (PRISMA) checklist [12]. Our study was registered prospectively with INPLASY (INPLASY202420102; https://doi.org/10.37766/inplasy2024.2.0102 accessed on 23 February 2024).

### 2.1. Eligibility Criteria

The literature we deemed eligible for inclusion included case reports and cohort or case–control studies that described cases with SCT who were prenatally diagnosed. The types of fetal surgeries included were classified as open surgery or percutaneous interventions; percutaneous interventions were defined as procedures to shrink the tumors, such as laser ablation, radiofrequency ablation, thermocoagulation, embolization, and sclerosis.

Articles and studies that described procedures such as tumor cyst puncture, amnioreduction, and fetal transfusion during SCT management were excluded, as their intention was not to reduce tumor size.

### 2.2. Information Sources

The sources used for data collection were PubMed and Google Scholar. Papers referencing sacrococcygeal teratomas were viewed from the inception of these databases until 15 September 2023.

### 2.3. Search Strategy

We reviewed the literature to compare the indications for surgical intervention and the prognoses of SCT cases following open fetal surgery or percutaneous intervention, as well as papers discussing the survival rates of fetuses that did not have any fetal intervention.

The search was conducted using the following terms: “sacrococcygeal teratoma” AND “fetal intervention” OR “fetal surgery” OR “open surgery” OR “in utero treatment” OR “fetal therapy” OR “RFA” OR “laser ablation” OR “ablation” OR “coagulation” OR “thermocoagulation” OR “radiofrequency” OR “embolization” OR “coiling” OR “sclerosis” OR “alcohol”).

Cases without fetal intervention were located using the term “fetal sacrococcygeal teratoma,” and the timeframe of the papers included ranged from 2014 to 15 September 2023. The reference lists of relevant articles were reviewed manually, with duplicate cases excluded, and eligible studies were added to the results from the electronic literature search.

### 2.4. Selection Process

As previously stated, papers that prescribed the diagnosis of SCT in the fetal period were the focus of this review, and only papers falling into the categories of case reports and cohort or case–control studies were deemed eligible. The screening process was completed by two independent reviewers (*H.K.* and *L.S.*).

### 2.5. Data Extraction

Using a standardized spreadsheet, data extraction from the included articles was performed independently by the two authors who completed the data selection, *H.K.* and *L.S.* The extracted information had the first author’s name, year of publication, country of origin, study design, patient demographic data, perinatal variables (as defined in the outcome measures), and type of intervention undergone.

In cases where there was an overlap or duplication of patients between studies, the details for both studies were included for review. The overlap of study populations was assessed based on the authors, the institution where the study was performed, and the year of data collection and publication.

### 2.6. Outcome Measures

The indications and outcomes of open surgery and percutaneous interventions for SCT cases and between cases with and without intervention were compared.

Procedure-related variables, such as tumor size, the presence of fetal hydrops, fetal heart failure, polyhydramnios, gestation weeks, and both intervention and delivery, were obtained and compared between the survivor cases and non-survivor cases. Tumor size was determined by extracting the volume directly from an excised tumor or by measuring the tumor in all three direction planes via imaging techniques such as ultrasound. In the cases where all three directions could not be determined by ultrasound, the following formula was applied: *tumor size (volume)=* 4/3*πa^3^ or* 4/3π*ab(a+b)/2, in which the radius in one direction is donated by (a) and the mean of the radii in two directions (a, b).

### 2.7. Statistical Analysis

The chi-square test was used to analyze categorical variables, and the *t*-test or Mann–Whitney test was used to analyze continuous variables as required. Significance was defined as *p* < 0.05. Statistical analysis was performed using R Ver 4.1.0.

## 3. Results

### 3.1. Study Selection

**Figure 1:**
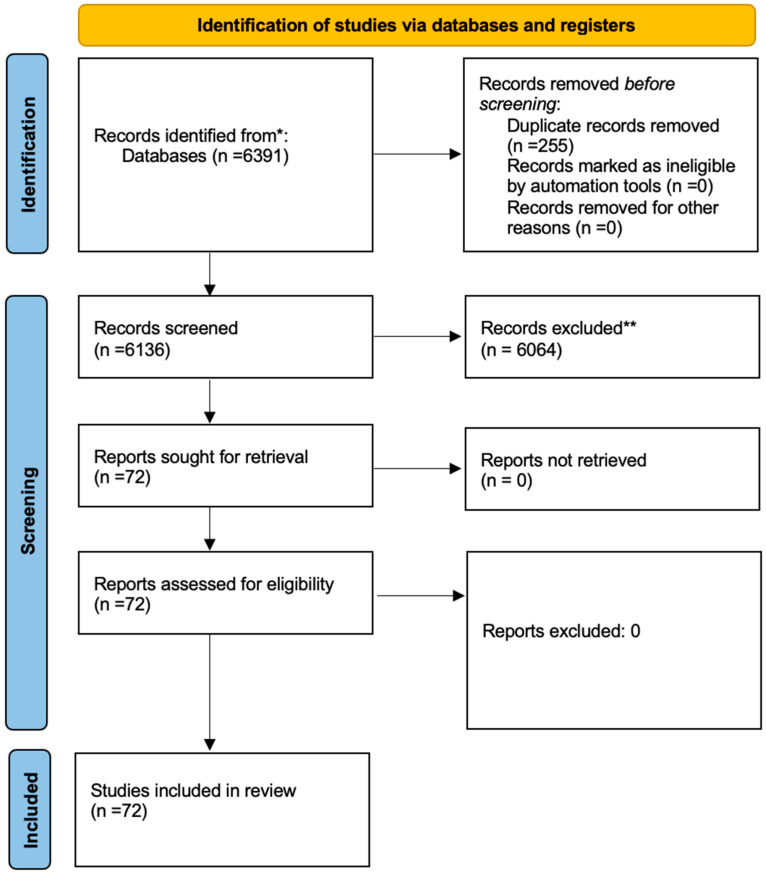
PRISMA flowchart of the study selection. * The search was conducted using the following terms: “sacrococcygeal teratoma” AND (“fetal intervention” OR “fetal surgery” OR “open surgery” OR “in utero treatment” OR “fetal therapy” OR “RFA” OR “laser ablation” OR “ablation” OR “coagulation” OR “thermocoagulation” OR “radiofrequency” OR “embolization” OR “coiling” OR “sclerosis” OR “alcohol”). Furthermore, we searched for cases without fetal intervention using the term “fetal sacrococcygeal teratoma” from 2014 to 15 September 2023. ** We excluded because of the following reasons: (1) target diseases are different, (2) not in the target period, (3) no detailed data, (4) languages other than English.

A total of 6391 articles were retrieved from the electronic search, with a data breakdown of 541 citations yielded from PubMed and 5850 from Google Scholar. Following the literature review relevant to our topic, 255 papers were excluded as these were duplicates, leaving a total of 6136 abstracts and titles screened. A flowchart highlighting the process from our initial literature search to literature inclusion is shown in Figure 1.

Ultimately, out of the 6136 papers screened, 72 papers were analyzed [1,11,13,14,15,16,17,18,19,20,21,22,23,24,25,26,27,28,29,30,31,32,33,34,35,36,37,38,39,40,41,42,43,44,45,46,47,48,49,50,51,52,53,54,55,56,57,58,59,60,61,62,63,64,65,66,67,68,69,70,71,72,73,74,75,76,77,78,79,80,81,82]; all papers included in our final screening comprised case reports, case series, and cohort studies.

A total of 16 open fetal surgeries and 48 cases of percutaneous fetal intervention were identified, with the percutaneous interventions being divided into 23 laser ablations, 18 radiofrequency ablations (RFA), 5 cases of alcohol sclerosis, 1 case of coiling, and 1 case of thermocoagulation.

Ninety-three cases of SCT without any fetal intervention were also noted. These cases are reviewed and summarized in Table 1.

### 3.2. Study Characteristics

The characteristics of all cases are presented in Table 1. The treatment strategies depended on the policies of each facility at the time, and there were no fixed standards. In cases without fetal intervention, survival rates were significantly higher than in cohorts where surgery was the primary care method. The gestational week at delivery tended to be later. The rates of SCT complications, including fetal hydrops and fetal cardiac failure, were also lower without fetal intervention.

A total of 9 fetuses (56.2%) survived after open surgery for the fetal SCT (OS) group and 22 (45.8%) in the percutaneous intervention (PI) group (*p* = 0.568). The survival rates of each percutaneous intervention were 39% (laser ablation: 9/23), 67% (RFA: 12/18), 20% (alcohol sclerosis: 1/5), and 0% (coiling: 0/1 and thermocoagulation: 0/1) (*p* = 0.160). Although the gestational weeks at intervention tended to be earlier in the percutaneous intervention group than in the open surgery group (*p* = 0.061), there were no significant differences in gestational weeks at delivery between the two groups (*p* = 0.863). Tumors were more sizable in the open surgery group than the percutaneous intervention group (*p* = 0.044). There was no significant difference in the rate of fetal heart failure between the two groups (*p* = 1.000), but the rate of fetal hydrops was significantly higher in the open surgery group than the percutaneous intervention group (*p* < 0.001) (Table 1, Table 2 and Table 3).

Table 4 and Table 5 show data comparing cases where fetal survival was compared to cases without. In both groups, there was no significant difference in the presence of fetal hydrops before intervention (OS: *p* = 0.475, PI: *p* = 0.107). The gestational age at delivery was earlier in non-survivors than in survivors, even excluding fetal demise cases (OS: *p* = 0.033, PI: *p* = 0.006). In the open surgery group, the tumor size of survivors was significantly smaller than that in the non-survivors (*p* = 0.026). In the percutaneous intervention group, tumor sizes tended to be more prominent in the poorer prognostic cases compared to their cohorts, although these differences were not statistically significant (*p* = 0.371).

In the non-surgical groups, fetal hydrops and fetal cardiac failure were observed in 8/75 cases (10.7%) and 9/74 cases (12.2%), respectively. Despite the lack of iatrogenic intervention, the overall survival rate was 71%; five out of eight of cases (62.5%) with fetal hydrops and six out of nine cases with fetal cardiac failure.

Table 6 shows the data comparing survival cases and non-survival cases in no-intervention group. While there were no significant differences in the presence of fetal hydrops and cardiac failure between survivors and non-survivors (*p* = 0.111, *p* = 0.156), the cases with polyhydramnios in fetuses that perished were relatively high (*p* < 0.001).

In circumstances where fetal hydrops developed, survival rates varied between open surgeries and percutaneous procedures, with survival occurring in 7/14 cases (50%) of open surgery and in 4/15 cases (26.7%) of percutaneous intervention; 5/8 (62.5%) of the no-intervention cases with fetal hydrops survived.

When reviewing papers that discussed the development of cardiac failure in utero, the survival rates in the groups reviewed, open surgery, percutaneous intervention, and conservative management, were as follows: 4/6 cases (66.7%), 12/27 cases (44.4%), and 6/9 cases (66.7%). The median of gestational weeks at delivery in fetuses with cardiac failure was 29 weeks (26–35) in the open surgery group, 28 weeks (17–38) in the percutaneous intervention group, and 30 weeks (21–35) in the group that had no intervention.

## 4. Discussion

In this review, the data revealed 16 cases of open fetal surgery, 48 cases of percutaneous fetal intervention, and 93 cases without fetal intervention. Our data collection highlighted no significant difference in survival rate between the open surgery group and the percutaneous intervention group, with a total survival of 56.2% in open surgery cases and 45.8% in percutaneous intervention cases. In each group, the mean gestational age at delivery was significantly earlier in the babies who were submitted to an intrauterine procedure and died compared to those who were submitted to intra-uterine fetal surgery and survived.

SCT can be diagnosed in the prenatal period, usually by ultrasound examination during the second trimester, but the mortality rate of fetal SCTs with prenatal diagnosis is higher when compared to those diagnosed postnatally, probably because nowadays, those small SCTs may not be diagnosed prenatally [83,84]. Furthermore, fetal SCTs with hydrops or fetal cardiac failure are associated with worse outcomes, especially increased perinatal mortality rate. The perinatal mortality rate of fetal SCTs with hydrops or fetal cardiac failure is greater than 50%, which is higher than the mortality rate of fetal cases without them [19,35,83,85]. SCTs with highly vascularized tumors and rapid growth are associated with increased risk of progression to fetal hydrops or fetal cardiac failure, and, therefore, associated with increased risk of mortality. Another complication of large SCTs with increased vascularity is the increased risk of fetal anemia due to intra-tumoral hemorrhage, which can also progress to elevate cardiac outputs and fetal cardiac dysfunction [1,20].

If the fetus has hydrops or fetal cardiac failure, intrauterine fetal interventions are indicated with the objective of improving the perinatal survival rate [11,16,18,22,23,86]. In general, based on the literature, fetal interventions are performed in approximately 13% of fetal SCTs in order to improve prognoses when SCT is associated with fetal hydrops, or cardiac dysfunction [35]. The purpose of fetal intervention is to prevent progression to fetal hydrops, fetal cardiac failure, or the rapid growth of the tumor. Open fetal resection of the tumor and percutaneous fetal intervention (laser ablation, RFA, thermocoagulation, or embolization) are different techniques and methods used as fetal interventions for fetuses with SCTs [11,16,18,22,23,86]. So far, there has been no evidence that one option is better than the other regarding the objectives described above [11,33,34,86,87].

Therefore, the present systematic review and meta-analysis evaluated which procedure could have better results. In this study, there was no significant difference in the survival rates considering different types of intrauterine fetal intervention, that is, 56.2% in open surgery cases and 45.8% in percutaneous intervention cases. Although open surgery tended to be chosen in cases deemed more severe, we found that the gestational weeks at delivery in more extensive surgery and percutaneous intervention cases were almost identical. In both procedures, the mean gestational age of delivery was earlier in non-survival cases than survival cases, even excluding fetal demise cases, regardless of the presence of the fetal hydrops, fetal cardiac failure, or polyhydramnios. Although it is difficult to determine the best method of fetal intervention based on this review, the management to prevent premature delivery after fetal intervention should be considered very important and more research should be focused on this subject.

On the other hand, in cases without fetal intervention, the gestational age at delivery was also significantly earlier in non-survival cases than in survival cases. The presence of polyhydramnios in non-survival cases was significantly more frequent than in those that survived after fetal intervention. This could suggest that we should closely monitor polyhydramnios to prevent premature delivery.

Our study did not aim to compare outcomes between patients submitted to fetal interventions and those with prenatal expectant management in fetuses with SCT, since fetal interventions are indicated for severe forms of SCT when hydrops or fetal cardiac dysfunction is present, while prenatal expectant management was performed in fetuses with SCT without those complications. In addition, there was no randomized controlled trial comparing these two groups (fetal interventions vs. non-fetal interventions) as some ethical questions may be considered. Our study, therefore, focused on comparing different methods of fetal intervention in fetuses with severe SCT (associated with hydrops and/or fetal cardiac dysfunction). In addition, it seems that the different methods of fetal intervention for severe SCT are chosen based on the surgeon’s experience. There are no data in the literature that provide possible indications for different fetal therapeutic options for severe SCTs.

An important strength of our review is that it highlights fetal outcomes when presenting with prevalent complications associated with SCT, such as fetal hydrops and/or fetal cardiac failure. In addition, this is the largest systematic review on this subject at the moment.

Our study has some limitations mainly regarding the heterogeneity of the study. Additionally, there was no randomized controlled trial or prospective study conducted comparing different techniques, especially with non-fetal intervention, since fetal sacrococcygeal tumors are quite rare. And the cases of good prognostic outcomes tend not to be reported, especially in cases without fetal intervention. Therefore, our study was not able to evaluate the effectiveness of fetal surgery (any type) compared to fetuses without fetal intervention. The literature lacks these types of studies. The literature compares outcomes with old studies that reported the natural history of SCTs with hydrops and/or cardiac failure. Furthermore, the extent of tumor resection, considerations based on pathologic diagnosis, and long-term outcomes including neurodevelopment or oncological prognoses, were not considered and have few data.

Therefore, it is necessary in the future to accumulate and study cases and consider which patients need fetal intervention and which patients are good candidates for open or percutaneous surgery. To this end, the focus should extend to long-term prognosis, including infants without fetal interventions.

## 5. Conclusions

As part of the group of rare diseases, sacrococcygeal teratomas can present with a myriad of consequences and complications in a developing fetus. Our review has highlighted the importance of identifying these conditions promptly. While further investigations of SCTs remains warranted, our data emphasize that there are different therapeutic options to treat in utero fetuses with SCT associated with hydrops and/or cardiac failure with similar outcomes. However, our systematic review and meta-analysis show that there is no significant difference in perinatal outcomes considering different types of intrauterine fetal intervention. In our opinion, since this is a rare condition, further large prospective multicenter database studies are warranted in order to investigate the impact of different types of intrauterine fetal surgeries for large SCTs with fetal hydrops or fetal cardiac dysfunction.

## Figures and Tables

**Table 1:** Characteristics of all cases.

	Open Surgery (OS) *n* = 16	Percutaneous Intervention (PI) *n* = 48	*p*-Value	Without Intervention (NI) *n* = 93
Survival cases	9 (56.2%)	22 (45.8%)	0.645	66 (71.0%)
Gestational age at delivery (weeks)	28.0 (21–35)	28.5 (17–38)	0.863	35.0 (19–40)
Gestational age at intervention (weeks)	24.0 (21–27)	22.0 (17–21)	0.061	N/A
Tumor size (cm^3^)	481 (161–936)	130 (10–1932)	0.044	442.7(1.4–8181.2)
Fetal hydrops (*n* = 59)	14/16 (87.5%)	15/43 (34.9%)	<0.001	8/76 (10.5%)
Fetal cardiac failure (*n* = 40)	6/7 (85.7%)	27/33 (81.8%)	1.000	9/75 (12.0%)

Data are presented as medians (ranges) or numbers (%).

**Table 2:** Summary of cases of SCT who underwent open surgery.

Ref.	N	GW at Diagnosis (Weeks)	Tumor Size (cm^3^)	Hydrops	Heart Failure	Polyhydramnios	GW at Fetal Intervention (Weeks)	Fetal Transfusion	GW at Delivery (Weeks)	Indication of Preterm Delivery	Outcome
Adzick (1997) [13,14]	1	20	350	yes	no	yes	26	no	29	Preterm labor	alive
	3	N/A	670	yes	yes	yes	23	yes	27	Preterm labor, pPROM	NND
		N/A	341	yes	yes	yes	21	yes	31	Preterm labor, pPROM	alive
		N/A	590	no	yes	yes	25	no	27	Preterm labor, pPROM	alive
Graf (1998, 2000) [13,15,16,17,18]	3	N/A	N/A	yes	N/A	N/A	24	no	26		NND
		N/A	N/A	yes	N/A	N/A	27	no	28		NND
		N/A	N/A	yes	N/A	N/A	23	yes	28	Preterm labor	alive
Westerburg (2000) [19]	7	18	372	yes	N/A	no	N/A	N/A	34		not alive
		17	376	yes	N/A	yes	N/A	N/A	21		not alive
		19	936	yes	N/A	yes	N/A	N/A	26		not alive
		21	900	yes	N/A	no	N/A	N/A	25		not alive (mirror syndrome)
		20	195	yes	N/A	yes	N/A	N/A	28		alive
		25	161	yes	N/A	no	N/A	N/A	30		alive
		17	335	yes	N/A	no	N/A	N/A	27		alive
Cass (2021) [1,20]	2	N/A	N/A	yes	yes	N/A	23	N/A	35		alive
		N/A	N/A	yes	yes	N/A	N/A	N/A	35		alive

GW, gestational weeks; pPROM, preterm premature rupture of membranes; NND, neonatal death.

**Table 3:** Summary of cases of SCT who underwent percutaneous intervention.

Ref.	N	GW at Diagnosis (Weeks)	Tumor Size (cm^3^)	Hydrops	Heart Failure	Polyhydramnios	GW at Fetal Intervention (Weeks)	Type of Intervention	Fetal Transfusion	GW at Delivery (Weeks)	Indication of Preterm Delivery	Outcome
Hecher (1996) [21]	1	16	62	no	no	yes	20	laser vascular ablation	yes	37		alive
Paek (2001) [22]	4	20	381	yes	yes	no	20	RFA (entire tumor)	no	N/A		IUFD
		21	330	yes	yes	no	21	RFA (large vessels)	no	28	Hydrops	alive
		19	118	no	yes	no	19	RFA (large vessels)	no	31	pPROM, NRFS	alive
		18	309	no (yes after RFA)	yes	yes	22	RFA (large vessels)	yes	25		TOP
Lam (2002) [23]	1	13	141	no	yes	yes	18	Thermocoagulation	no	24		IUFD
Ibrahim (2003) [24]	1	18	118				20	RFA	no	32	Placenta abruption	alive
Perrotin (2005) [25]	1	13	N/A	yes	yes	yes	27	Alcohol sclerosis	yes	29	pPROM	alive
Benachi (2006) [26]	1	N/A	>10 cm	N/A	N/A	N/A	N/A	Coiling	no	24		IUFD
Makin (2006) [27]	7	N/A	N/A	yes	N/A	N/A	N/A	Laser vascular ablation	no	32		alive
		N/A	N/A	yes	N/A	N/A	N/A	Laser vascular ablation	no	24		IUFD
		N/A	N/A	yes	N/A	N/A	N/A	Laser vascular ablation	no	32		IUFD
		N/A	N/A	yes	N/A	N/A	N/A	Laser vascular ablation	no	28		NND
		N/A	N/A	yes	N/A	N/A	N/A	Alcohol sclerosis	no	27		IUFD
		N/A	N/A	yes	N/A	N/A	N/A	Alcohol sclerosis	no	32		NND
		N/A	N/A	yes	N/A	N/A	N/A	Alcohol sclerosis	no	27		NND
Grethel (2007) [28]	4	N/A	N/A	no	N/A	N/A	N/A	RFA	N/A	N/A		alive
		N/A	N/A	no	N/A	N/A	N/A	RFA	N/A	N/A		alive
		N/A	N/A	no	N/A	N/A	N/A	RFA	N/A	N/A		NND
		N/A	N/A	no	N/A	N/A	N/A	RFA	N/A	N/A		NND
Ruano (2009) [29]	1	23	1030	yes	yes	yes	24	Laser ablation	no	N/A		IUFD
Ding (2010) [30]	1	19	17	no	yes	yes	22	Laser vascular ablation	no	29	Antepartum hemorrhage from previa	alive
Lee (2011) [31]	6	22	113	no	yes	(50%)	25	RFA	(1/6)	33	Preterm labor	alive
		20	119	no	no		23	RFA with cyst aspiration		26	pPROM	alive
		16	11	no	no		20,22	RFA		27	pPROM	NND
		22	21	no	yes		31	RFA		35	pPROM	alive
		29	278	no	no		31	RFA		35	Preterm labor	alive
		22	258	no	no		23,30	RFA with T-A shunt		35	Preterm labor	alive
Usui (2012) [32]	1	N/A	N/A	N/A	N/A	N/A	N/A	RFA	no	N/A		alive
Van Mieghem (2014) [11]	5	26	817	no	yes	yes	26	Laser ablation (large superficial vessels)	no	26	NRFS (during laser)	NND
		21	879	no	yes	yes	22	RFA	yes	22		IUFD (during RFA)
		26	1327	no	yes	yes	26	RFA	no	27	Preterm labor	alive
		17	114	no	yes	no	17	Laser + coiling	no	17		IUFD
		26	1191	no	yes	no	26	Laser + coiling	yes	28		alive
Sananes (2015) [33]	5	21	(1.93 cm^3^/g)	yes		yes	24	Laser ablation (interstitial)	no	N/A		IUFD
		N/A	(1.89 cm^3^/g)	yes	yes	yes	21	Laser ablation	no	23	HELLP	NND
		N/A	(2.37 cm^3^/g)	yes	no	yes	22	Laser ablation	no	32	Preterm labor	alive
		N/A	980	no	yes	yes	21	Laser ablation	21,24,28	34	pPROM, preterm labor	alive
		N/A	452	yes	yes	yes	23	Alcohol sclerosis	23	25	Mirror syndrome	NND
Litwinska(2018) [34]	7	N/A	62	no	yes	no	20	Laser ablation	no	38		alive
		N/A	58	no	yes	no	21,24	Laser ablation	no	29	Preterm labor	alive
		N/A	92	no	yes	no	23,27	Laser ablation	no	30	Preterm labor	alive
		N/A	74	no	yes	no	20,22	Laser ablation	no	24		IUFD
		N/A	54	no	yes	no	19,20	Laser ablation	no	31	Preterm labor	NND
		N/A	39	no	yes	no	19	Laser ablation	no	29	pPROM	NND
		N/A	589	no	yes	yes	23,24	Laser ablation	no	25	Mirror syndrome	NND
Van Heurn (2021) [35]	1	21	1932	N/A	yes	N/A	25	Interstitial laser coagulation (not complete)	no	27		NND
Sosa (2021) [36]	1	21	N/A	N/A	yes	N/A	24,27,30	Laser sclerosis	N/A	34	pPROM, preterm labor	NND

GW, gestational weeks; RFA, Radio Frequency Ablation; IUFD, Intrauterine Fetal Death; pPROM, preterm premature rupture of membranes; NRFS, non-reassuring fetal status; TOP, termination of pregnancy; HELLP, hemolysis, elevated liver enzymes, and low platelets syndrome; NND, neonatal death.

**Table 4:** The data on open surgery cases.

	Survival Cases	Non-Survival Cases	*p*
Tumor size (cm^3^)	338 (161–590)	641.5 (372–936)	0.026
Fetal hydrops	7/9 (77.8%)	7/7 (100%)	0.475
Fetal cardiac failure	4/5 (80%)	2/2 (100%)	1.000
Polyhydramnios	4/6 (66.7%)	4/6 (66.7%)	1.000
Gestational age in weeks at fetal intervention	24 (21–26)	24 (23–27)	0.692
Gestational age in weeks at delivery	29 (27–35)	26 (21–34)	0.033

Data are presented as medians (ranges) or numbers (%).

**Table 5:** The data on percutaneous intervention cases.

	Survival Cases	Non-Survival Cases	*p*
Tumor size (cm^3^)	118 (10–1327)	345 (11–1932)	0.371
Fetal hydrops	4/20 (20%)	11/23 (47.8%)	0.107
Fetal cardiac failure	12/17 (70.6%)	15/16 (93.8%)	0.175
Polyhydramnios	6/12 (50%)	9/14 (64.3%)	0.692
Gestational age in weeks at fetal intervention	22.5 (19–31)	22.0 (17–26)	0.195
Gestational age in weeks at delivery	32 (26–38)	26 (17–34)	<0.001
Without fetal demise (*n* = 36)	32 (26–38)	27 (23–34)	0.006

Data are presented as medians (ranges) or numbers (%).

**Table 6:** The data on cases without fetal intervention.

	Survival Cases	Non-Survival Cases	*p*
Tumor size (cm^3^)	314.2 (1.4–8181.2)	467.2 (20.9–4188.8)	0.376
Fetal hydrops	5/63 (7.9%)	3/12 (25%)	0.111
Fetal cardiac failure	6/62 (9.7%)	3/12 (25%)	0.156
Polyhydramnios	15/59 (25.4%)	10/13 (76.9%)	<0.001
Type 1 or 2	49/63 (14.3%)	19/21 (90.5%)	0.336
Gestational age at delivery	37 (27–40)	28 (19–36)	<0.001
Without fetal demise (*n* = 87)	27 (27–40)	29 (21–36)	<0.001

Data are presented as medians (ranges) or numbers (%).

## Data Availability

The data presented in this study are available on request from the corresponding author.
